# The impact of tobacco tax/law implementation on pancreatic cancer mortality in Mexico, 1999–2015

**DOI:** 10.3332/ecancer.2018.ed85

**Published:** 2018-10-10

**Authors:** Eduardo Hernández-Garduño

**Affiliations:** Department of Education and Health Research, Instituto de Seguridad Social del Estado de México y Municipios (ISSEMyM), Toluca, México 50080, Mexico

**Keywords:** pancreatic cancer, mortality, tobacco tax/law, smoking prevalence, pancreatic cancer risk factors

## Abstract

Among the multiple aetiologies identified for pancreatic cancer (PC), cigarette smoking and diabetes are considered “moderate risk factors”. Analysis of PC mortality trends is important as changes in incidence and mortality of this tumour can be partially attributable to changes in smoking patterns. A recent Mexican study examined PC mortality trends and showed a favourable trend from 2000 to 2014 [1]. However, the impact of new tobacco tax/laws which were implemented in Mexico in 2007/2008 was not assessed in this study. In this re-analysis we assessed their impact on PC mortality and found a non-statistically significant trend from 1999 to 2008 - however, PC mortality statistically decreased from 2008 with an annual percent change or APC of −1.27, −1.23 and −1.17 in both sexes, females and males respectively, p < 0.05. These declines are likely resulting in part from new tobacco tax/laws which are likely contributing to the decrease over time of smoking prevalence and environmental tobacco smoke exposure.

## Introduction

PC is among the cancers with the poorest prognosis with a 5-year survival rate of only 5 to 7%. Among modifiable risk factors, smoking is well established and one of the most important with an average increased risk for ever-smokers of 70% and an estimated population attributable fraction of 11–32% [2]. One of the studies on the mechanism linking tobacco and PC performed on mice exposed for 20 weeks to cigarette smoke showed an increase of ADM formation and accelerated PanIN development in conjunction with decreased number of myeloid-derived suppressor cells and increase in the number of M2 macrophages and dendritic cells in the pancreas, accelerating PC progression [3].

**Figure 1.**
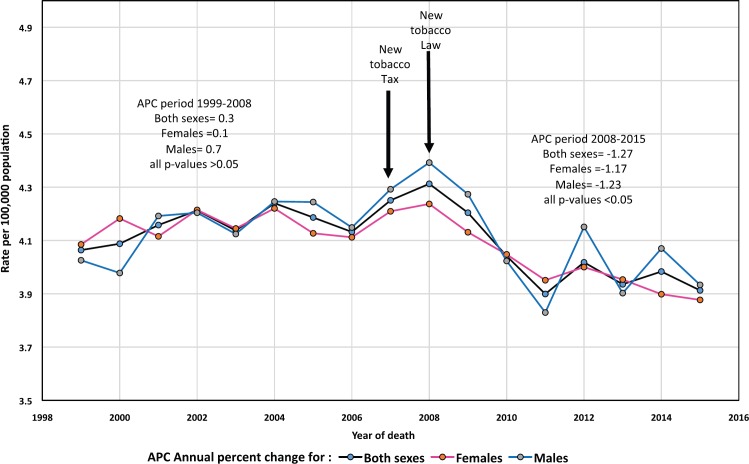
Age-standardised mortality rates of pancreatic cancer, all ages Mexico 1999–2015.

Two recent epidemiological studies from Spain and Mexico analysed PC mortality temporal trends. The Spanish study [4] showed an unfavourable increasing trend in men from 1975 to 1986 (APC = 4.1) and from 1986 to 2012 (APC = 1.1). Corresponding APCs for women were 3.6 and 1.4 respectively. The slower increasing rate in the last period could be associated with decrease of smoking prevalence in men since the late eighties while in women prevalence rose until the late nineties and then slightly decreased [5] possibly explaining the higher APC estimate in women in the second period. Contrary to these findings, a recent countrywide Mexican study showed a favourable decreasing trend in PC mortality from 2000 to 2014 with APCs for the whole period of −1.08, −0.89 and −0.87 for both sexes, females and males respectively [1]. Figure 1 of that Mexican study showed a higher rate of decline from 2008 onward but the authors did not determine whether this apparent change in trend was statistically significant. In this re-analysis we employed joinpoint trend analysis [6] to determine this. This is important because a new tobacco tax and law in Mexico were implemented in 2007 and 2008 respectively [7,8].

## Pancreatic cancer mortality analysis in Mexico since the implementation of tobacco tax/law in 2007/2008

Mortality data for this re-analysis was obtained from the same source (INEGI) [9] as the previous study [1]. We increased the study time period from 1999 to 2015 because data became available. The number of PC deaths (ICD-10th code C25) and crude rates were calculated by year, sex and by 5-year age groups based on population growth estimates published by the “Consejo Nacional de Población” [10]. Age-standardised mortality rates (ASMR) were then calculated according to the World (WHO 2000-2025) Standard Population [11].

There were a total of 57,169 pancreatic deaths during the 17-years study period (53.0% females, annual mean deaths of 3,362). The median ASMR of the 17-year period was 4.1 for both sexes, females and males (Table 1) with a favourable trend of ASMR-APC in the whole period (−0.33, 95% CI −0.6 - −0.1 P = 0.023) however, an increasing non statistically significant trend was found from 1999 to 2008 (APC = 0.32, 95% CI −0.1–0.8, P = 0.10) but it became favourable from 2008 to 2015 in both sexes (APC = −1.27, 95% CI −1.9 - −0.6, P = 0.001) with slightly higher rate of decline in females (APC: −1.23) than males (−1.17), p < 0.05, Figure 1.

## Relevance of the temporal changes of pancreatic cancer mortality and tobacco smoking patterns

The decline in PC mortality over time may be partly explained by the reduction of smoking prevalence. According to national addiction surveys for the years 2002 and 2016-2017 [12,13], the overall prevalence of current smokers for Mexican adults aged 18 to 65 years decreased from 27% in 2002 to 20.1% in 2016-2017 (25% reduction), from 42.3% to 31.3% in males (26.0% reduction) and from 15.1% to 9.8% (35.1% reduction) in females. The higher reduction rate of smoking prevalence in females may explain the higher rate of decline in females’ APC.

The favourable decreasing trend in PC from 2008 coincided with the implementation of an increase in cigarette taxes in 2007 [7] and legislation protecting nonsmokers from second hand smoke in 2008 [8]. Even the overall prevalence of second-hand smoke (defined in national surveys as involuntary exposure to cigarette smoke in nonsmokers with specific questions for exposure in households, work, pubs, schools, restaurants and public transportation) in all adults did not change from 2002 (22.5%) to 2016-2017 (22.6%), it did in females, 30.7% to 21.3% respectively with 30% reduction [12,13]. This may have also contributed to the higher rate of APC decline in females from 2008.

Our recent study showed a reduction in lung cancer ASMR’s median in Mexicans aged ≥ 40 years from 15.8 (1999-2008) to 13.6 (2008-2014) in females (14.0% reduction) and from 38.7 to 28.3 in males (27% reduction) respectively [14].This can also be attributed to implementation of tobacco tax/law and the decrease of smoking prevalence over time though the smaller reduction of female’s ASMR median is attributable to the still high prevalence of wood smoke exposure from cooking which was 40.5% in 2012/2013 in rural areas [15]. Long term wood smoke exposure has been associated with lung cancer in nonsmoking women in Mexico [16]. Future studies are needed to determine whether long-term wood smoke exposure is associated with PC.

Besides smoking, diabetes is a known risk factor associated with PC. We have recently shown that diabetes mortality decreased in Mexican females age 20 to 79 years (APC = −0.9) after the implementation of “Seguro Popular” (SP) in 2004 [17] which is health insurance offered primarily for the poor offering free diagnostics, hospitalisation, treatment and counseling for diabetes among other diseases. Better diabetes control likely explains the decrease in diabetes mortality in females and contributes to the higher rate of decline in females’ PC compared to males. A positive dose response relationship between post load glycemia and PC mortality has been reported [18].

## Conclusion

The decrease in PC mortality in this analysis and of lung cancer mortality in our previous study is likely attributable partly to decreasing smoking prevalence and exposure to second-hand smoke over time in Mexico. The statistically significant decreasing trend in PC mortality after 2008 confirms the effectiveness of new tobacco tax/law in Mexico. This link demonstrates the importance for assessing impacts of changes in health policy on specific health outcomes.

## Conflicts of interest

The author has no conflicts to report.

## Funding

The author did not receive any funding for this work.

## Figures and Tables

**Table 1.** Distribution of pancreatic cancer deaths by year of death and gender, Whole country of Mexico, 1999–2015.

Year of death	Both sexes:		Females:		Males:	
	N=	ASMR	N=	ASMR	N=	ASMR
1999	2,558	4.1	1,354	4.1	1,204	4.0
2000	2,654	4.1	1,432	4.2	1,222	4.0
2001	2,783	4.2	1,456	4.1	1,327	4.2
2002	2,927	4.2	1,549	4.2	1,378	4.2
2003	2,964	4.1	1,569	4.1	1,395	4.1
2004	3,129	4.2	1,655	4.2	1,474	4.2
2005	3,185	4.2	1,673	4.1	1,512	4.2
2006	3,241	4.1	1,713	4.1	1,528	4.1
2007	3,444	4.3	1,814	4.2	1,630	4.3
2008	3,607	4.3	1,895	4.2	1,712	4.4
2009	3,635	4.2	1,912	4.1	1,723	4.3
2010	3,618	4.0	1,943	4.0	1,675	4.0
2011	3,598	3.9	1,955	4.0	1,643	3.8
2012	3,821	4.0	1,993	4.0	1,828	4.2
2013	3,863	3.9	2,092	4.0	1,771	3.9
2014	4,039	4.0	2,125	3.9	1,914	4.1
2015	4,103	3.9	2,196	3.9	1,907	3.9
Total	57,169		30,326		26,843	
Median		4.1	1,814	4.1	1,630	4.1
ASMR = Age-standardised mortality rates per 100,000 population						
